# Two-Way Short Message Service (SMS) Communication May Increase Pre-Exposure Prophylaxis Continuation and Adherence Among Pregnant and Postpartum Women in Kenya

**DOI:** 10.9745/GHSP-D-19-00347

**Published:** 2020-03-30

**Authors:** Jillian Pintye, Zoe Rogers, John Kinuthia, Kenneth K. Mugwanya, Felix Abuna, Harison Lagat, Joseph Sila, Valarie Kemunto, Jared M. Baeten, Grace John-Stewart, Jennifer A. Unger

**Affiliations:** a Department of Global Health, University of Washington, Seattle, Washington, USA.; b Department of Biobehavioral Nursing and Health Informatics, University of Washington, Seattle, WA, USA.; c Department of Obstetrics/Gynecology, Kenyatta National Hospital, Nairobi, Kenya.; d Department of Epidemiology, University of Washington, Seattle, Washington, USA.; e Department of Medicine, University of Washington, Seattle, Washington, USA.; f Department of Pediatrics, University of Washington, Seattle, Washington, USA.; g Department of Obstetrics/Gynecology, University of Washington, Seattle, Washington, USA.

## Abstract

**Introduction:**

We evaluated a 2-way short message service (SMS) communication platform to improve continuation of pre-exposure prophylaxis (PrEP) for HIV prevention among Kenyan women who initiated PrEP within routine maternal child health (MCH) and family planning clinics.

**Methods:**

We adapted an existing SMS platform (Mobile WACh [mWACh]) to send PrEP-tailored, theory-based SMS and allow clients to communicate with a remote nurse. Women who did not have HIV and who were initiating PrEP at 2 MCH/family planning clinics in Kisumu County, Kenya, from February to October 2018, were offered enrollment into the mWACh-PrEP program; SMS communication was free. We evaluated acceptability, satisfaction, and implementation metrics. In a pre/postevaluation, we compared PrEP continuation at 1-month postinitiation among women who initiated PrEP in the period before (n=166) versus after mWACh-PrEP implementation, adjusting for baseline differences.

**Results:**

Of the 334 women who were screened for enrollment into the mWACh-PrEP program; 193 (58%) were eligible and of those, 190 (98%) accepted enrollment. Reasons for ineligibility (n=141) included no phone access (29%) and shared SIM cards (25%). Median age was 25 years (interquartile range=22–30), and 91% were MCH clients. Compared to women who initiated PrEP in the month before mWACh-PrEP implementation, women who enrolled in mWACh-PrEP were more likely to return for their first PrEP follow-up visit (40% vs. 53%; adjusted risk ratio [aRR]=1.26; 95% confidence interval [CI]= 1.06, 1.50; *P*=.008) and more likely to continue PrEP (22% vs. 43%; aRR=1.75; 95% CI=1.21, 2.55; *P*=.003). Among those who returned, 99% reported successful receipt of SMS through the mWACh-PrEP system and 94% reported that mWACh-PrEP helped them understand PrEP better. Concerns about PrEP use, how it works, and side effects accounted for the majority (80%) of issues raised by participants using SMS.

**Conclusions:**

Two-way SMS expanded support for PrEP and opportunities for dialogue beyond the clinic and enabled women to ask and receive answers in real time regarding PrEP, which facilitated its continued use.

## Abstract (precis)

We evaluated a 2-way short messaging service (SMS) communication platform to improve continuation of pre-exposure prophylaxis (PrEP) for HIV prevention among Kenyan pregnant and postpartum women who initiated PrEP within routine maternal child health and family planning clinics. SMS increased support for PrEP, provided opportunities for dialogue beyond the clinic, and enabled women to ask and receive answers in real-time, which facilitated continued PrEP use.

## INTRODUCTION

Young women in sub-Saharan Africa have one of the highest HIV incidence rates globally.^1^ This high HIV incidence persists during pregnancy and breastfeeding,^2^ and there is evidence that HIV acquisition risk increases by more than 2-fold during pregnancy and the postpartum period.^3^ Pregnant women who become acutely infected with HIV account for an estimated 26% of all vertical HIV transmissions.^4^^,^^5^ To prevent HIV acquisition during pregnancy and reach elimination of vertical transmission, the World Health Organization recommends offering oral tenofovir-based pre-exposure prophylaxis (PrEP) to pregnant women who do not have HIV in high-burden settings.^6^

Data from Kenya estimated that HIV incidence among pregnant and postpartum women is 2.31/100 person-years.^7^ Programmatic PrEP delivery to pregnant and postpartum women in maternal child health (MCH) clinics is ongoing in Kenya,^8^^,^^9^ and other countries are planning PrEP implementation within MCH settings.

Although a majority of pregnant and postpartum women with HIV risk factors accepted PrEP when offered within routine MCH settings in Kenya,^9^ more than 50% discontinued PrEP within 30 days of initiation. Mobile health (mHealth) tools can be used to educate clients, provide reminders for visits and medications, improve communication between health care workers and clients, and improve self‐efficacy, all potentially leading to better medication adherence outcomes.^10^^–^^13^ Studies are ongoing to enhance PrEP continuation and adherence using mHealth approaches in diverse populations including men who have sex with men, transgender women, and adolescent girls who are not pregnant.^14^^–^^17^ To date, no PrEP adherence intervention studies have targeted pregnant or postpartum women who have unique considerations for PrEP use. Qualitative studies have found that pregnant women who use PrEP are highly motivated to protect their infants from HIV, though some have concerns about whether PrEP affects their infant,^18^ and postpartum women have challenges remembering to take PrEP pills during the complex transition to motherhood.^18^ Therefore, mHealth strategies tailored to pregnant and postpartum women who use PrEP are needed.

To support PrEP continuation and adherence among pregnant and postpartum women, we adapted the mobile communication platform, Mobile WACh [mWACh]—named for the University of Washington's Global Center for Integrated Health of Women, Adolescents, and Children—^19^^–^^21^ using 2-way short message service (SMS) between participants and remote nurses. We conducted a mixed-methods evaluation within a programmatic PrEP delivery setting to assess implementation metrics and evaluate PrEP continuation and adherence outcomes among women PrEP initiators enrolled in the SMS program.

## METHODS

### Program and Setting

The PrEP Implementation for Young Women and Adolescents (PrIYA) Program was a 2-year implementation project in Kusumu County, Kenya, a region where adult HIV prevalence is 19.9% (up to 28% among pregnant women).^22^^–^^24^ The program was designed to reach adolescents and young women at high risk for HIV acquisition through integrated delivery of PrEP within routine MCH and family planning systems.^9^ Conducted in collaboration with the Kisumu County Department of Health and Sanitation and the National AIDS and STI Control Programme, PrIYA was first implemented in 16 facilities (11 public, 4 faith-based, and 1 private) followed by a PrEP mentorship program in 20 additional sites. The 16 highest volume (based on monthly number of new antenatal care ANC clients) facilities in Kisumu County were selected to be in the PrIYA Program. The current evaluation focuses on the implementation of a 2-way SMS intervention among a subset of participants in the PrIYA Program. The SMS intervention was conducted at 2 public-sector sites purposively selected based on the highest monthly enrollment of new PrEP clients.

In the PrIYA Program, 40 program-supported nurses were trained on PrEP delivery per national guidelines as previously described.^25^ Briefly, nurses screened women who did not have HIV for behavioral risk factors, including male partner HIV status and willingness to consider PrEP. Behavioral risk factors were assessed using a standardized risk assessment tool.^8^ Women who wanted to initiate PrEP and were medically eligible received same-day PrEP and were scheduled for a 1-month follow-up visit.^9^

### mWACh-PrEP Program Evaluation Design

This mixed-methods evaluation of the SMS communication program for supporting PrEP adherence and continuation had 3 primary aims: (1) describe implementation metrics (e.g., eligibility, acceptability, satisfaction, and utilization); (2) compare frequency of PrEP continuation and self-reported adherence before and after the introduction of the SMS program; and (3) identify issues and concerns among new PrEP initiators by qualitatively analyzing transcripts of SMS conversations.

### mWACh-PrEP Program Development

Our team previously developed mWACh, a user-friendly bidirectional interactive SMS platform, that enables efficient communication between women attending MCH clinics and nurses.^19^ The mWACh platform sends timed preprogrammed SMS messages that clients can respond to and allows clients to send messages with questions or concerns. A trained nurse-counselor receives and responds to messages from women, providing a mechanism for women to interact with nurses in real-time and an opportunity for medication adherence support.^9^ mWACh has been shown to be feasible, acceptable, and effective in improving MCH outcomes.^20^^,^^21^

**Figure:**
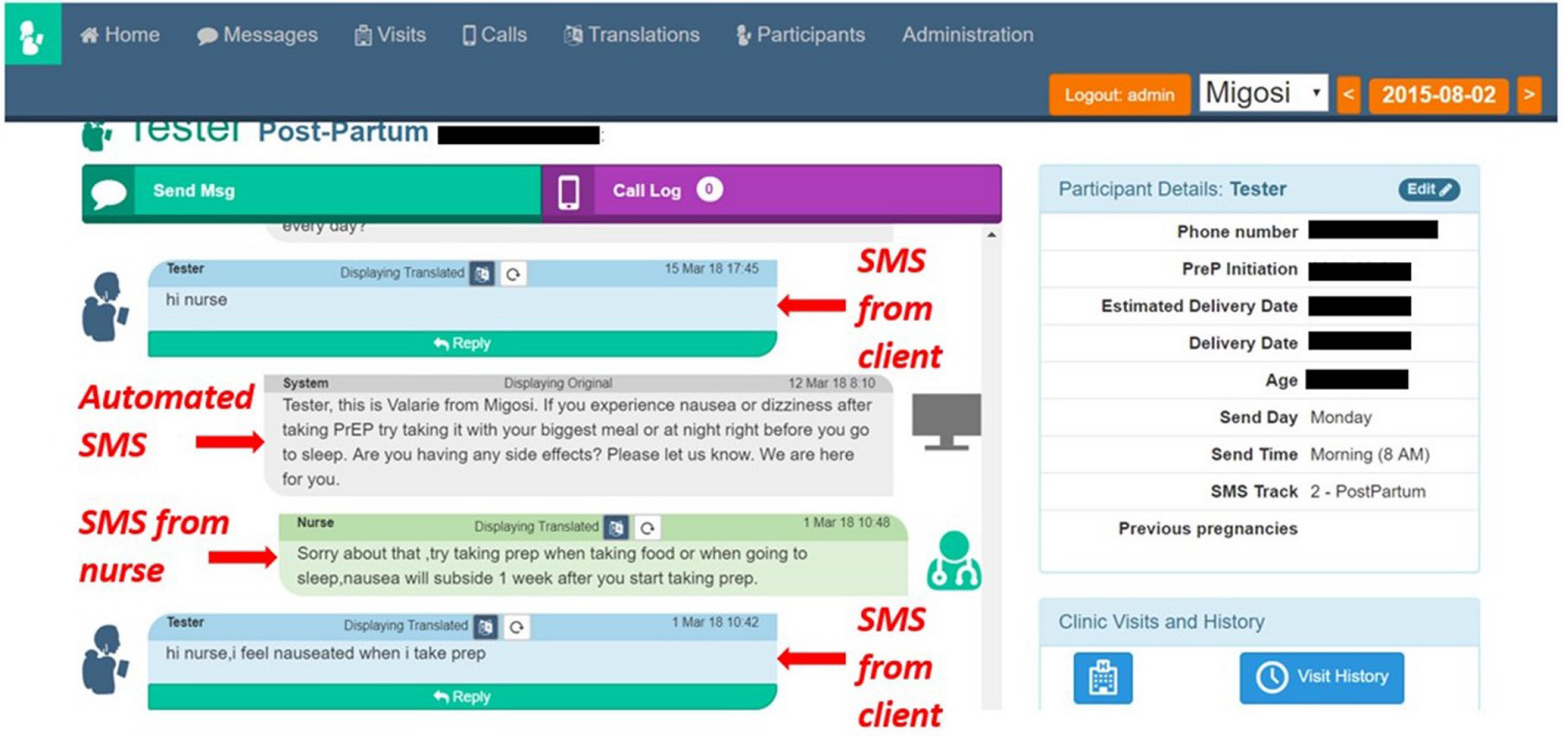
Interface of mWACh-PrEP System With Mock Data© 2019 Jillian Pintye

We adapted the mWACh platform to send weekly PrEP-tailored, theory-based SMS and to allow MCH and family planning clients who initiate PrEP to communicate with a nurse about their individual needs. SMS messages incorporated behavioral theory-informed counseling framed in the informational-motivational-behavioral skills model, specifically tailored to women receiving PrEP in the context of MCH/family planning services (Box). The intervention was field-tested by PrIYA nurses with more than 6 months of experience delivering PrEP to MCH and family planning clients before implementation. During field-testing, we elicited feedback from PrEP providers on the utility, frequency, and content of SMS messages to refine the tool. The final message bank was translated and back translated from English to Kiswahili and Dhuluo (a prevalent local language in western Kenya). PrIYA nurses were trained on using the mWACH-PrEP system and responding to SMS messages from clients based on behavioral counseling principles including informational-motivational-behavioral skills, motivational interviewing, and positive reinforcement. We also developed and tested quality assurance/quality control systems that reviewed and discussed responses to SMS with medical and research teams in Kenya and Seattle to ensure consistency and accuracy of nurses' responses to women.

BOX **Example SMS From Automated Message Bank and Behavioral Theory Adapted for PrEP**

Motivational Interviewing (e.g., goal-oriented action plans)

{name}, this is {nurse} from {clinic}. It can be difficult to take medications every day especially if you are trying to be discrete. Many people ask a friend to help remind them, set a timer on their phone, or take it with a meal. You can also put it in a different container you can carry with you in private. How do you remember to take your medication? Do you have any challenges taking it every day?

Theory of Planned Behavior (e.g., perceived behavioral control)

{name}, this is {nurse} from {clinic}. Side effects from PrEP affect each person differently. Most side effects lessen after the first few weeks of use, once the body is used to the medication. Please let us know if you are having any side effects. We can help you manage them or know if it is okay to continue.

Health Belief Model (e.g., perceived barriers)

{name}, this is {nurse} from {clinic}. You are doing a great job taking care of yourself. PrEP is very effective at preventing you from getting HIV if you take it every day. It also helps prevent HIV infection to your baby if you are pregnant. If you miss too many doses it may not work. Are you having any challenges taking the medication?

Social-Cognitive Theory (e.g., positive reinforcement)

Good job coming in for your visit! You will receive weekly SMS to help support taking your medication. Please SMS back and tell us any questions or concerns you have. Please tell us if you need assistance with your prevention medication. If you have any challenges or stop PrEP, please let us know.

### Program Procedures

From February to October 2018, we approached women on the same day they initiated PrEP at the 2 selected MCH/family planning clinics and offered them participation in the mWACH-PrEP program. Women were eligible if they initiated PrEP that day, had a functioning cellphone in-hand that they did not share with anyone, and had an active SIM card on the Safaricom network (Kenya's largest network provider). Reasons for ineligibility were captured. We did not exclude women who expressed confidentiality concerns related to their PrEP use or content of the SMS. All women were free to decline enrollment and welcome to stop receiving SMS at anytime.

Women registered into the mWACh-PrEP platform indicated their preferences for message delivery including a preferred name for messaging, language (English, Kiswahili, or Dholuo), and day of the week and time for SMS delivery. All automated push messages included participant nickname, clinic, and nurse name, an educational message or actionable advice targeting PrEP adherence and continuation and/or MCH/family planning topics, and a question related to the content. SMS topics included adherence encouragement, PrEP efficacy and safety, self-efficacy for prevention of HIV, support for potential PrEP side effects, behavioral skills (tips for remembering PrEP medications), and visit reminders. During enrollment, the program nurse explained that replies to the automated SMS questions were voluntary, though women were enrouraged to reply. Women were also encouraged to send SMS with their concerns or questions whenever they arose. The program nurse was available to answer SMS during normal business hours on weekdays. All messaging was free of charge to the participant using a reverse billed short code. SMS were sent from enrollment until December 2018. Participants could voluntarily and autonomously exit the program by texting “STOP,” which would end all platform communication.

### Data Management and Analysis

All SMS communication was conducted through our custom web application designed for 3‐way communication between the automated push system, program staff, and participants. SMS data were downloaded biweekly by program data managers to track SMS outages and data on implementation activity (e.g., new enrollments, sucessful automated SMS delivery, participant responses, voluntary exits). When a participant sent an unprompted question or sent a reply to an automated message that was not in English, program nurses translated the SMS into English within the system daily to ensure interpretation consistency and allow for quality assurance/quality control. We defined utilization using 2 metrics: responding to automated push messages and sending an unprompted question or concern using SMS.

Program nurses administered questionnaires to women enrolled in mWACh-PrEP at routine 1-month PrEP follow-up visits to assess satisfaction using a series of items on a 5-point Likert scale (e.g., I would recommend the SMS program to other women who use PrEP; strongly agree, agree, neutral, disagree, strongly disagree). Closed-ended items assessed self-reported utilization experiences with the platform (e.g., I took action based on the nurse's advice; yes/no). We also abstracted PrEP indicators from the Kenya Ministry of Health client encounter form that included data on attendance of a PrEP follow-up visit, self-reported adherence (number of missed PrEP doses in the past month), and PrEP refills. We defined PrEP continuation as confirmed dispensation of a PrEP refill at an attended follow-up visit. PrEP discontinuation was defined as no PrEP refill or no attendance at a follow-up visit. Data from PrEP follow-up visits were abstracted for all women who initiated PrEP in the month before implementation of the mWACh-PrEP program and from all women who were screened for mWACh-PrEP enrollment.

We used descriptive statistics to summarize acceptability, satisfaction, and utilization indicators. We used Chi-squared tests for proportions and Kruskall-Wallis tests for continous measures to compare baseline demographic and behavioral characteristics of women who were eligible and enrolled in the mWACh-PrEP program with (1) women who initiated PrEP in the month before implementation of mWACh-PrEP and (2) women who were screened for enrollment but were ineligible/declined. We compared PrEP continuation and self-reported adherence among women who enrolled in mWACh-PrEP with women in the other 2 groups using Chi-squared tests and multivariate Poisson regression models with robust error variance, an approach used when the outcome prevalence is not rare (e.g., >10%).^26^^,^^27^ Final multivariate Poisson models were adjusted for age and marital status because these characteristics were significantly different between women who enrolled in the mWACh-PrEP program and those who initiated PrEP before mWACh-PrEP implementation. All statistical analyses were performed by using StataSE 15.0 (StataCorp, College Station, TX). Statistical comparisons were 2-sided and were considered significant at the *P*<.05 level.

### Qualitative Analysis

At the completion of the mWACh-PrEP program, transcripts of SMS conversations were analyzed using a modified constant comparative approach. Our primary goal was to identify issues raised by women who initiated PrEP through unprompted questions or concerns sent to nurses using the mWACh-PrEP system. We developed an initial codebook based on a review of a subset of transcripts and input from nurses who responded to participants' messages, and the codebook was iteratively refined. SMS transcripts were transferred into Microsoft Excel for data management. All transcripts were coded by 2 research team members (ZR and JP), and coding disagreements were resolved through discussion. Key issues raised by women who enrolled in mWACh-PrEP were identified by reading transcripts to identify similarities and differences across conversations, and codes were subsequently organized within thematic categories to identify trends. We also selected representative quotations pertaining to each main category.

Our primary qualitative analysis goal was to identify issues that women who initiated PrEP raised through unprompted messages.

### Human Subjects Considerations

Protocols were reviewed and approved by the Kenyatta National Hospital-University of Nairobi Ethics Research Committee and University of Washington Human Subjects Review Committee. In addition, approval was obtained from the Kisumu County Department of Health and administrators in respective health facilities. Women provided informed consent for all activities.

## RESULTS

**FIGURE 1:**
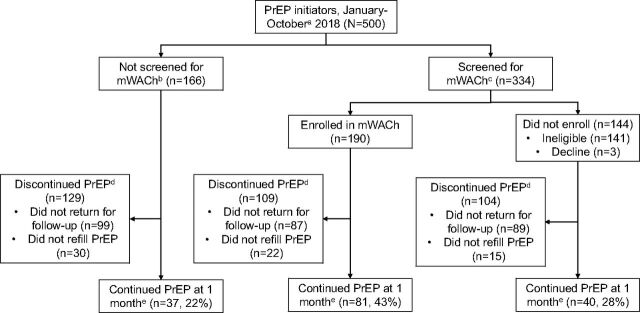
Enrollment of Women in mWACh Program at PrEP Initiation Visits, Kusumu County, Kenya Abbreviations: mWACh, Mobile WACh; PrEP, pre-exposure prophylaxis. ^a^ From 2 sites that participated in the mWACh-PrEP program. ^b^ PrEP initiators who were not screened for mWACh-PrEP initiated PrEP in January 2018 before mWACh-PrEP implementation, which began in February 2018. ^c^ PrEP initiators who were screened for mWACh-PrEP initiated PrEP between February and October 2018. ^d^ PrEP discontinuation was defined as either not returning for a follow-up visit or not refilling a PrEP prescription at a follow-up visit. ^e^ PrEP continuation was defined as attending a follow-up visit and refilling a PrEP prescription.

Overall, 334 women were screened for participation in the mWACH-PrEP program (Figure 1); 193 (58%) were eligible and 190 (98%) of eligible women enrolled. Reasons for ineligibility (n=141) included not having a phone (28%) or Safaricom SIM card (15%) in the clinic that day, sharing SIM cards (24%), using a network other than Safaricom (15%), having a broken phone (12%) or other phone issues (3%). Among the 3 (2%) women who declined, 2 feared intimate partner violence and 1 felt the program was unecessary because she did not anticipate adherence issues. Among women who enrolled in the mWACh-PrEP, 48% were 24 years old or younger, 91% were MCH clients, and 9% were family planning clients; 89% were married (Table 1). Almost half (47%) of women who enrolled reported having a male partner who did not have HIV, 10% had a known partner with HIV, and 44% had a male partner who did not know his HIV status. Among pregnant women who enrolled (n=73), the median gestational age was 25 weeks (interquartile range [IQR]=20–28).

**TABLE 1.** Characteristics of Women Screened for mWACH-PrEP and Women Who Initiated PrEP Before mWACh-PrEP,^a,b^ by Enrollment, Kusumu County, Kenya

	Screened for mWACh			Initiated PrEP Before mWACh (n=166)	
	Enrolled (n=190)	Ineligible or Declined^a^ (n=144)	*P* Value^b^		*P* Value^c^
Age, years, median (IQR)	25 (22–30)	23 (20–27)	<.001	24 (21–28)	.006
Age category, No. (%)					
<18 years	3 (1.6%)	9 (6.3%)	.04	11 (6.6%)	.06
18–24 years	89 (46.8%)	80 (55.6%)		77 (46.4%)	
25–29 years	46 (24.2%)	28 (19.4%)		47 (28.3%)	
30–34 years	38 (20.0%)	18 (12.5%)		22 (13.3%)	
≥35 years	14 (7.4%)	9 (6.3%)		9 (5.4%)	
Client recruitment clinic, No. (%)					
Antenatal care	78 (41.1%)	63 (43.8%)	.84	80 (48.2%)	.19
Postnatal care	95 (50.0%)	70 (48.6%)		78 (47.0%)	
Family planning	17 (9.0%)	11 (7.6%)		8 (4.8%)	
Married, No. (%)	171 (90.0%)	125 (86.8%)	.36	134 (81.2%)	.02
Male partner HIV status, No. (%)					
HIV-negative	89 (46.8%)	55 (38.2%)	.04	75 (45.5%)	.81
HIV-positive	19 (10.0%)	8 (5.6%)		14 (8.5%)	
Unknown	823 (43.2%)	81 (56.3%)		76 (46.1%)	
Gestational age, years, median (IQR)	25 (20–28)	26.5 (20–32.5)	.25	24.5 (20–32)	.51
First antenatal care visit, No. (%)	24 (34.3%)	16 (27.6%)	.42	38 (52.1%)	.03
Behavioral risk factors (last 6 months), No. (%)					
Had sex without a condom	187 (98.4%)	144 (100.0%)	.13	164 (98.8%)	.77
Exchanged sex for money or other favors	0 (0.0%)	2 (1.4%)	.10	1 (0.6%)	.28
Diagnosed with or treated for a sexually transmitted infection	1 (0.5%)	2 (1.4%)	.41	0 (0.0%)	.35
Forced to have sex against will	5 (2.6%)	4 (2.8%)	.93	8 (4.8%)	.27
Experienced intimate partner violence	4 (2.1%)	3 (2.1%)	.99	8 (4.8%)	.16

Abbreviations: mWACh, Mobile WACh; PrEP, pr-exposure prophylaxis.

a Overall, 3 women were eligible for mWACh-PrEP and declined participation; 141 women were ineligible.

b Chi-sqared tests for proportions or Kruskal-Wallis tests for continuous measures, comparing women who were eligible and enrolled with women who were ineligible/declined among those screened for mWACh-PrEP.

c Chi-sqared tests for proportions or Kruskal-Wallis tests for continuous measures, comparing women who were eligible and enrolled with women who initiated PrEP in the month before mWACh-PrEP implementation.

Women who were eligible and enrolled were slightly older than women who were ineligible or declined enrollment (median age 25 years olds and 23 years, respectively; *P*<.001). Compared to women who initiated PrEP in the period before mWACh-PrEP, eligible and enrolled women were also slightly older (median age 24 years old and 25 years, respectively; *P*=.006), more frequently married (81% and 90%, respectively; *P*=.02), and more frequently attending first ANC visits, if pregnant (33% and 52%, respectively; *P*=.03). There were no other differences in demographic and behavioral characteristics between women who were eligible and enrolled and women who were either ineligible/declined or who initiated PrEP before mWACh-PrEP (Table 1).

### Acceptability and Satisfaction

Overall, 100 of 190 (53%) women who enrolled returned for a follow-up visit at a median of 29 days (IQR=28–40) since PrEP initiation. Almost all (99%) reported successful receipt of SMS messages through the mWACh-PrEP system. Among the 99 women who received messages, 72 (73%) women reported consulting by SMS with the nurse and, of those, 47 (66%) reported continuing PrEP because of the nurse's advice (Supplemental Tables). Most women (94%) reported that the SMS helped them understand PrEP better and 89% reported that the SMS helped them adhere to PrEP. Almost all (95%) would recommend mWACh-PrEP to other women who use PrEP, and 95% would also use the program again, if offered (Supplemental Tables).

Most women reported that the SMS helped them understand PrEP better and helped them adhere to PrEP.

### PrEP Continuation and Adherence

Among the 166 women who initiated PrEP in the month before mWACh-PrEP implementation, 67 (40%) attended a PrEP follow-up visit compared to 101 (53%) of women who enrolled in mWACh-PrEP (adjusted risk ratio [aRR]=1.26; 95% confidence interval [CI]=1.06, 1.50; *P*=.008). Compared to women who initiated PrEP in the month before mWACh-PrEP implementation, women who enrolled in mWACh-PrEP were almost twice as likely to continue PrEP after adjustment for age and marital status (22% vs. 43%; aRR=1.75; 95% CI=1.21, 2.55; *P*=.003). Among women who returned for follow-up, 74 (73%) of women who enrolled in mWACh-PrEP self-reported high PrEP adherence (<1 missed pill/week) compared to 37 (55%) of women who initiated PrEP before mWACh-PrEP (aRR=1.35; 95% CI=1.28, 1.41; *P*<.001). Compared to women in a contemporaneous cohort who were ineligible or declined enrollment, women enrolled in mWACh-PrEP were also more likely to return for a follow-up visit (38% vs. 52%, respectively; *P*=.02) and continue PrEP (28% vs. 43%; *P*=.005); among those who attended follow-up visits, self-reported high adherence was similar across those contemporaneous groups (67% vs. 73%, respectively *P*=.42).

### Participant Utilization of mWACH-PrEP

Full transcripts from SMS conversations were available and analyzed for 170 of the 190 (89%) women who enrolled in the mWACh-PrEP program. Among 170 women with analyzed transcripts, 97 (57%) ever responded to the automated messages. Median duration of enrollment in the mHealth program was 24 weeks (IQR=17–31). Frequency of responding to automated messages was highest at week 1 (29%) and substantially lower by week 6 (14%) (Figure 2a). The median time to response cessation was 6 weeks (IQR=1–13) among women who ever responded.

**FIGURE 2a:**
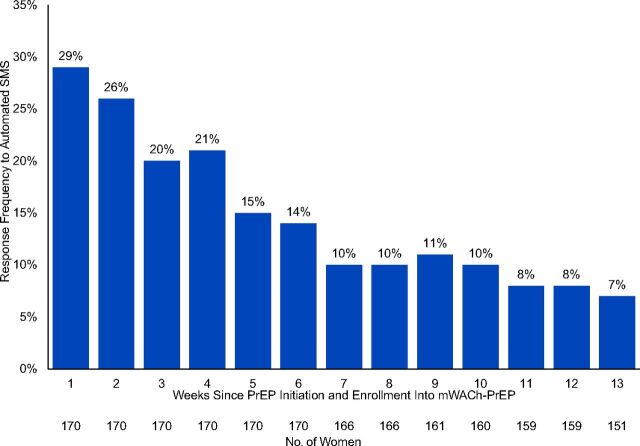
Women's Response Frequency to Automated SMS Over Time Since PrEP Initiation and Enrollment in mWACh-PrEP Program, Kusumu County, Kenya^a^ Abbreviations: mWACh, Mobile WACh; PrEP, pre-exposure prophylaxis; SMS, short message service. ^a^ Full SMS transcripts were available and analyzed for 170/190 (90%) of women who enrolled into mWACh-PrEP.

Overall, 74 of the 170 (44%) participants ever sent unprompted questions or concerns to the nurse using SMS during follow-up, and a total of 183 unprompted SMS were received and answered by nurses. The median number of unprompted questions/concerns sent per client was 2 (IQR=1–3). Seven major topics were raised by participants in their SMS queries (Figure 2b). PrEP continuation and discontinuation was the most frequently raised topic overall (27%). These messages included queries about how long one must continue PrEP use, if stopping and restarting PrEP is possible, and self-reporting PrEP discontinuation among participants who did not return for follow-up visits (Table 2). Side effects (24%) were the next most frequent topic of unprompted questions/concerns. These messages included requests for advice on dealing with side effects, confirmation of whether symptoms experienced (e.g., nausea, vomiting, weakness) were normal with PrEP use, and clarification of whether and when symptoms would eventually subside. Participants also raised issues about the logistics of PrEP use (16%), including where/how to get refills and what to do if doses were missed. Participants also sought clarifications about PrEP (13%) including how PrEP works, how it is different from drugs used to treat HIV, and whether men could also take PrEP. The remaining 20% of unprompted questions and concerns sent by participants and answered by nurses were not PrEP-related. These messages included concerns regarding HIV risk (3%), queries regarding MCH or family planning issues in general (4%), and other topics like allergic reactions to other medications or relationship concerns (13%).

**FIGURE 2b:**
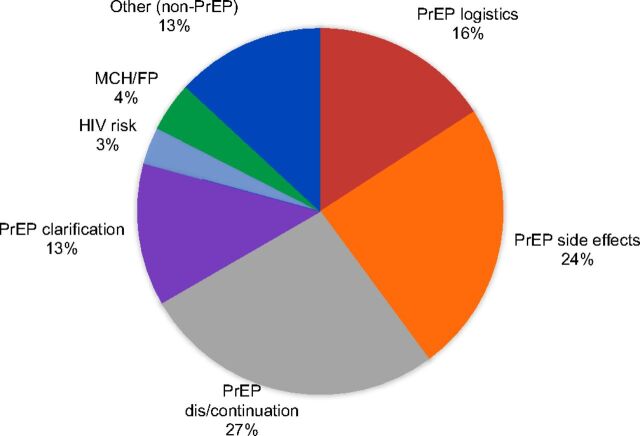
Frequency Distribution of Unprompted Question and Concern Topics Sent by Women Who Initiated PrEP to Remote Nurses Using the mWACh-PrEP Platform, Kusumu County, Kenya (N=183)^a^ Abbreviations: mWACh, Mobile WACh; PrEP, pre-exposure prophylaxis; SMS, short message service. ^a^ Overall, 170 participants who initiated PrEP were enrolled in the mWACh-PrEP program had full transcripts available for analysis and 74 (44%) ever sent an unprompted question to a remote nurse during follow-up. Remote nurses received and responded to 183 unprompted questions in total from 74 women.

**TABLE 2.** Representative Quotations Pertaining to Each Main Topic of Questions or Concerns and Correponding Responses, mWACh-PrEP Platform^a^

Topic	Unprompted Question or Concern Sent by Participants by SMS	Nurse's Response to Participant (Time to Response From SMS Receipt)
PrEP dis/continuation	Thanks, nurse, for the concern. Just want to know the duration of taking PrEP? Because in my case, we are a discordant couple.	Hello {Name}. It is important that you continue taking PrEP until your partner's viral load is suppressed. You can incorporate condoms during sexual intercourse to protect you further. We encourage that you freely share health information related to your partners care. (14 hours; participant sent SMS in the evening, nurse responded the next morning)
	Hi, I separated with my husband because of mistrust. He felt ill and was taken to the hospital, where he tested HIV-negative and malaria-positive. Should I continue using this drug? I am not a sex worker.	Hello {Name}. You can continue taking your medication. But should you desire to discontinue, please come back to our clinic for further advice. I would also like to tell you that PrEP is not ONLY used by sex workers, but also those who feel they are at risk of becoming infected. (3 hours)
	Hi {Nurse's name}. If I'm abstaining from sex and I also stay alone, should I continue to use this drug?	Hello {Name}. If you feel you are no longer at risk of being infected, you may stop PrEP. However, you should continue taking the drug 1 pill daily for 28 days from the last risk period. (1 hour)
Side effects	After taking it (PrEP), and I vomit once a day.	Nausea and vomiting is common in pregnancy and could also be a side effect of PrEP. Minor side effects like nausea and vomiting are manageable. Avoiding fatty food, eating smaller meals, and limiting spicy foods may help. PrEP minor side effects subside after a short period of time. (20 minutes)
PrEP clarification	Hey, is it true that PrEP is a type of ARV? They say it is given to HIV+ and HIV- individuals.	Hello {Name}. PrEP is classified as one of the antiretroviral medications used to protect someone from HIV/AIDS. PrEP medication is given to different people depending on the substantial risks involved. Discordant couples, unknown HIV status of your sexual partner, and individuals with multiple sexual partners are some of the people at risk who could be eligible to use PrEP. (2 hours)
	Does PrEP prevent someone from conceiving?	Morning {Name}. PrEP does not prevent someone from conceiving. It is safe for pregnancy as well. It can have side effects like headache, nausea, and vomiting that subsidize with time. (1 hour)
	I abide by what you people told me at the clinic, but some information I was told elsewhere is different. I heard it is not good to use this medication (PrEP) until I know my partner's status. Another issue is that PrEP is an antiretroviral medication. Can I get HIV/AIDS if I use PrEP medication for long periods?	Hello, {Name}. PrEP medication helps to prevent HIV/AIDS acquisition. Not knowing your partners status is one of the risks that may lead to an individual acquiring HIV/AIDS. It is important to know your partner's status because it helps to reduce the risk of HIV acquisition. In case you want to stop PrEP, it is advisable to come back to the clinic for discontinuation. (2 hours)
PrEP logistics	Hello, Nurse. I would like to ask if it is good to take my medications before taking my meals?	Hello, {Name}. It is good to take the medications after meals to avoid side effects such as nausea and vomiting. (10 minutes)
	Is it a must that I should take it (PrEP) at bedtime?	Hello, it is not a must to take at bedtime. PrEP can be taken one tablet daily either in the morning, afternoon or evening. It is also advisable to be strict on the time you take the medication. In case you decide its morning hours, then every day you should take it in the morning. (19 hours; participant sent SMS in the evening, nurse responded the next morning)
	In case I forget to take PrEP medication today and then I remember the next day, can I take the medicine then?	Hello, at that point in time when you remember to take medication, you should swallow 1 tablet. It is not advisable to swallow 2 tablets at once because you forgot taking the medication the previous day. You can set an alarm on your phone to act as a reminder when to take the medication. (20 hours; participant sent SMS in the evening, nurse responded the next morning)
HIV risk	I am inquiring if I have 2 partners, 1 has HIV and the other one is HIV-negative. If it happens that I have sex with the negative partner, can he get HIV because the other one is positive?	How are you, {Name}. You can't transmit HIV to him because you are negative and you are on PrEP taking them faithfully. Again, if your positive partner is taking antiretrovirals as prescribed, he can't transmit HIV to you…..However, you should also use condoms when you are meeting your negative partner because you don't know if he is having multiple partners. (4 hours)
MCH/family planning	Hello, I have been using Depo Provera for a long time until I stopped because of excess bleeding…I haven't experienced any of these side effects since I stopped using the injection. My monthly periods have resumed normally, 5 days, with 3 heavy days and lighter 2 days. Which method of family planning can I use without side effects?	Hello, I would kindly advise you to find time and come to the hospital so that we can talk more on the other methods of family planning, so that you can be able to decide and choose one of the family planning methods that you would want to use. (2 hours)
	Now is when the baby is breastfeeding too much because she had mouth rash, she was not feeding.	Hello {Name}. Breastfeeding is important for the baby because it helps in growth and development. Did you bring the child for review at the clinic regarding the oral thrush? When the baby has lesions in the mouth it can affect how the baby breastfeeds and feeding orally. (16 hours; participant sent SMS in the evening, nurse responded the next morning)
Other (not related to PrEP)	I have a problem when having sex with my husband with a condom. I experience bloating and abdominal pain. What can I do please? Should I stop having sex?	Hi {Name}. You can stop having sex, but I would like you to see a doctor for further management and instructions. (23 hours; participant sent SMS on the weekend, nurse responded on Monday)

a Some quotations have been modified from their original short message form to increase language clarity.

## DISCUSSION

In this mixed-methods evaluation of an mHealth tool within a programmatic PrEP delivery setting, we found very high acceptance (98%) among the subset of women who met inclusion criteria, an almost 2-fold greater early PrEP continuation, and higher self-reported adherence among women who were enrolled in mWACh-PrEP than those who initiated PrEP before mWACh-PrEP implementation. We also found high satisfaction with the mWACh-PrEP system and high utilization. Almost half (44%) of the women who enrolled had unprompted questions or concerns addressed using SMS, most of which were PrEP-related. To our knowledge, this is the first evaluation of an mHealth intervention for PrEP adherence targeting pregnant and postpartum women, and this communication platform addressed topics very specific to pregnancy and the postpartum period. As PrEP delivery within routine MCH/family planning settings expands, mHealth strategies tailored to pregnant and postpartum women may improve PrEP continuation and adherence in this unique population.

This is the first evaluation of an mHealth intervention for PrEP adherence targeting pregnant and postpartum women.

We previously reported that PrEP counseling before initiation in the PrIYA program lasted approximately 18 minutes per client.^25^ Women who initiated PrEP received this standardized counseling from nurses who were experienced with PrEP delivery, yet clarifications and concerns about the PrEP use and how PrEP works accounted for more than half (56%) of all unprompted issues raised by the participants. This indicated that many women who initiated PrEP left the clinic with concerns about PrEP use or that PrEP concerns may have arisen after they left the clinic that would otherwise go unaddressed without access to a remote nurse. Additionally, nearly one-quarter (24%) of issues raised by participants pertained to side effects. Pregnancy may amplify side effects associated with early PrEP use (e.g., nausea, vomiting, gastrointestinal alterations), and experiencing side effects is a leading cause of PrEP discontinuation in this population.^28^ mHealth applications have successfully facilitated monitoring and mitigation of medication side effects for other health conditions in low- and middle-income countries.^29^^,^^30^ Promoting self-management of PrEP side effects and empowering women by filling PrEP knowledge gaps could be a mechanism by which the mWACh-PrEP platform improved PrEP continuation in this study. We found diminished response to automated messages after 1 month, which suggests that a time-limited mHealth intervention may be sufficient to address concerns and support onboarding to sustained PrEP use.

Ongoing studies are testing mHealth tools that address various stages of the PrEP care continuum in diverse populations, including using mHealth to increase uptake^31^^,^^32^ and enhance adherence.^14^^–^^17^ We focused on early PrEP continuation and adherence among women who initiate PrEP within MCH/family planning because we previously found a steep drop-off in PrEP continuation and suboptimal PrEP adherence in this population. The mWACh platform was initially designed to address maternal and infant health concerns among pregnant and postpartum women.^19^ The adapted mWACh-PrEP system focused on PrEP use. Thus, our evaluation assessed short-term PrEP-specific outcomes and did not measure other MCH-related outcomes. Additionally, in this evaluation we did not assess provider time or other costs associated with implementation. Future studies should incorporate information beyond early PrEP outcomes, including impact on maternal/infant health and provider time and costs, to determine whether mWACh-PrEP is a cost-effective strategy within routine MCH/family planning systems delivering PrEP.

Although we found high acceptability in our evaluation, 42% of screened women were ineligible due to cell phone-related issues. The most frequent reason for ineligibility was not having a cell phone in the clinic; this was required to confirm registration was successful. Future iterations of the mWACh-PrEP platform could incorporate off-site registration options for clients to complete self-registration at a later time. Additionally, not having a SIM card compatible with the mWACh system was another major reason for ineligibility. We partnered with Safaricom for mWACh-PrEP as this is the largest network provider in Kenya. Inclusion of other network providers within the platform, which is feasible at a modest additional cost, would increase accessibility and reach.

### Limitations

Our evaluation has limitations. We did not collect information on mWACh-PrEP eligibility criteria (e.g., phone availability) during the period before mWACh-PrEP implementation. Therefore, our pre/postevaluation design produced less rigorous results than a randomized trial, and we may have unmeasured differences between our comparison groups that could influence effect size. However, this nonrandomized evaluation provides insight into how mHealth interventions work in a real-world context. Within a program setting, we found nearly a 2-fold increase in PrEP continuation among women who enrolled in the mWACh-PrEP compared to both women who initiated PrEP before mWACh-PrEP implementation and a concurrent cohort of women who were ineligible/declined.

We intentionally restricted our comparison group to only January 2018, the month immediately preceding mWACh-PrEP implementation. Several events during the period before January 2018 were beyond control of the program and limited the program's ability to consistently provide PrEP in a representative population. A national nurses strike in Kenya from June through November 2017 halted service delivery in public-sector facilities,^33^ and the response in Kisumu to the presidential election of August 2017 also impacted the health sector.^34^ January 2018 was the most comparable month in terms of PrEP provision and stability of service delivery within health facilities in Kisumu.

We did not offer incentives or reimbursement for enrollment into mWACh-PrEP and did not conduct any procedures beyond routine clinical care and satisfaction surveys, including intensive follow-up procedures or contact tracing. Additionally, we offered mWACh-PrEP enrollment to all PrEP initiators, regardless of partner HIV status or other behavioral HIV risk factors. Therefore, our results may be more representative of what could be expected if mWACh-PrEP were to be programmatically rolled out than a randomized trial or an approach targeted to only women who self-disclose high behavioral HIV risk.

We relied on routinely collected data to measure PrEP continuation and self-reported adherence without an objective biomarker for PrEP exposure, which could have introduced reporting bias or misclassification. Studies comparing self-reported PrEP adherence to objective drug levels among men who have sex with men, transgender women, and individuals in HIV-serodiscordant couples in real-world settings have found reasonably high concordance.^35^^–^^37^ Future evaluations among pregnant and postpartum women in African settings could incorporate biomarkers of PrEP exposure to confirm self-reported adherence. We only included women who newly initiated PrEP. Future evaluations could assess whether the mWACh-PrEP program improves adherence among women with a poor adherence history. Additionally, our satisfaction data are limited to women who returned for a follow-up visit and may not be representative of all women enrolled in the mWACh-PrEP program.

## CONCLUSION

In summary, we found very high acceptance of the mWACh-PrEP program among pregnant and postpartum women and improved early PrEP continuation and adherence among those enrolled compared to women who initiated PrEP before the mWACH-PrEP program. mWACh-PrEP extended the reach of the clinic and enabled clients to promptly address concerns about PrEP which, in turn, appeared to help them continue and/or adhere to PrEP. Women found a short period of SMS support helpful. It is plausible that this system could be readily scalable and cost-effective, potentially improving outcomes and offsetting costs from unused or misused medicines.

## Supplementary Material

19-00347-Pintye-Supplement.pdf

## Notes

**Peer Reviewed**

**First published online:** March 6, 2020

**Funding:** This study was funded by the U.S. National Institutes of Health (grants #R01HD094630 and #R01HD100201). The PrEP Implementation for Young Women and Adolescents (PrIYA) Program was funded by the U.S. Department of State as part of the DREAMS Innovation Challenge (grant# 37188-1088 MOD01), managed by JSI Research & Training Institute, Inc. The PrIYA Team was supported by the University of Washington's Center for AIDS Research (grant# P30 AI027757) and the Global Center for Integrated Health of Women, Adolescents, and Children (Global WACh). The opinions, findings, and conclusions stated herein are those of the authors and do not necessarily reflect those of the U.S. Department of State or JSI.
