# Supplementary material for: Two-Way Short Message Service (SMS) Communication May Increase Pre-Exposure Prophylaxis Continuation and Adherence Among Pregnant and Postpartum Women in Kenya
Source: Glob Health Sci Pract. 2020 Mar 30;8(1):55–67. doi: 10.9745/GHSP-D-19-00347 (PMC7108943; doi:10.9745/GHSP-D-19-00347)
Supplement: 19-00347-Pintye-Supplement.pdf [file 19-00347-Pintye-Supplement.pdf]

Supplement to: Pintye J, Rogers Z, Kinuthia J, et al. Two-Way Short Message Service (SMS) Communication May Increase Pre-Exposure Prophylaxis Continuation and Adherence Among Pregnant and Postpartum Women in Kenya. *Glob Health Sci Pract*. 2020;8(1). https://doi.org/10.9745/GHSP-D-19-00347

Supplemental Table 1. Outcomes Among Women Who Enrolled in mHealth System and Returned for PrEP Follow-Up Visit, Kusuma County, Kenya (n=100)[a]

mHealth Follow-Up Outcomes	No. (%)
Successfully received mHealth messages	
Yes	99 (99%)
No	1 (1%)
Consulted with nurse via SMS at any time (n=99)	
Yes	72 (73%)
No	21 (21%)
Unknown	6 (6%)
Reasons for consulting with nurse (n=72)	
PrEP	67 (93%)
Pregnancy/postpartum concerns	9 (13%)
Other	3 (4%)
Took action based on nurse's advice (n=72)	
No action taken	1 (1%)
Sought medical advice	8 (11%)
Continued PrEP	46 (64%)
Discontinued PrEP	12 (17%)
Other	3 (4%)
Unknown	2 (3%)
Consult was helpful (n=72)	
Yes	72 (100%)
No	0 (0%)
Reasons for not consulting nurse (n=21)	
I didn't think about it/I forgot	8 (38%)
It was during the night or weekend	0 (0%)
I was afraid to/didn't want to	7 (33%)
I never found it necessary	9 (43%)
I didn't find the SMS helpful in the past	0 (0%)
Experienced harm/negative reactions from partner about SMS (n=99)	
Yes	0 (0%)
No	96 (97%)
Refused to answer	1 (1%)
Unknown	2 (2%)
Client would like to continue receiving messages (n=99)	
Yes	82 (83%)
No	16 (16%)
Unknown	1 (1%)

Supplement to: Pintye J, Rogers Z, Kinuthia J, et al. Two-Way Short Message Service (SMS) Communication May Increase Pre-Exposure Prophylaxis Continuation and Adherence Among Pregnant and Postpartum Women in Kenya. *Glob Health Sci Pract*. 2020;8(1). https://doi.org/10.9745/GHSP-D-19-00347

Reason for discontinuing messages (n=16)	
Discontinued PrEP	16 (100%)
SMS are not helpful	0 (0%)
Not interested	1 (6%)
Experienced harm or negative reactions from partner	0 (0%)
Other	1 (6%)

Abbreviations: mHealth, mobile health; PrEP, pre-exposure prophylaxis; SMS, short message service.
[a] Time since PrEP initiation/mHealth enrollment (n=89), median 29 days, IQR=28–40.

Supplement to: Pintye J, Rogers Z, Kinuthia J, et al. Two-Way Short Message Service (SMS) Communication May Increase Pre-Exposure Prophylaxis Continuation and Adherence Among Pregnant and Postpartum Women in Kenya. *Glob Health Sci Pract*. 2020;8(1). https://doi.org/10.9745/GHSP-D-19-00347

Supplemental Table 2. Acceptability and Satisfaction Indicators of mHealth System Among Women Who Enrolled in mHealth System, Received Messages, and Returned for a PrEP Follow-Up Visit (n=99)[a]

**Acceptability and Satisfaction Indicators**	Strongly Disagree	Disagree	Neutral	Agree	Strongly Agree	No response/ refuse
The SMS helped me to understand more about PrEP.	5%	--	1%	49%	44%	--
The SMS helped me to deal with challenges of PrEP (side effects, obtaining drugs, disclosure).	5%	--	--	58%	36%	1%
The SMS helped me to decide about continuing or discontinuing PrEP.	5%	2%	2%	57%	33%	1%
The SMS helped me adhere to PrEP.	4%	1%	5%	58%	31%	1%
I enjoyed interacting with my nurse via SMS.	2%	3%	10%	46%	31%	7%
I had non-PrEP concerns (i.e., MCH, relationship, infant care, etc.) addressed via SMS.	1%	3%	7%	47%	30%	11%
I received the right amount of SMS (not too much, not too little).	5%	--	--	60%	34%	1%
I would recommend the SMS program to other PrEP users.	5%	--	1%	55%	39%	--
If offered again, I would enroll in the SMS program.	5%	--	--	52%	41%	2%

Abbreviations: MCH, maternal and child health; mHealth, mobile health; PrEP, pre-exposure prophylaxis; SMS, short message service.
[a] Overall, 100 mWACh-PrEP enrollees returned for a follow-up visit; 99/100 (99%) confirmed successful receipt of SMS messages and were included in the analysis of acceptability and satisfaction indicators.
